# Determination of Serum Lost Goodwill Target Proteome in Patients with Severe Traumatic Brain Injury

**DOI:** 10.1155/2015/183821

**Published:** 2015-09-27

**Authors:** Hongming Ji, Changchen Hu, Gangli Zhang, Jinrui Ren, Yihu Tan, Wenxiao Sun, Junwen Wang, Jun Li, Hongchao Liu, Ruifan Xie, Zhipeng Hao, Dongsheng Guo

**Affiliations:** ^1^Department of Neurosurgery, Shanxi Provincial People's Hospital, Taiyuan 030001, China; ^2^Department of Neurosurgery, Tongji Medical College, Huazhong University of Science and Technology, Wuhan 430030, China; ^3^Department of Neurosurgery, Liyuan Hospital, Tongji Medical College, Huazhong University of Science and Technology, Wuhan 430077, China; ^4^Department of Neurosurgery, Wuhan Central Hospital, Tongji Medical College, Huazhong University of Science and Technology, Wuhan 430014, China

## Abstract

This study investigates the biokinetics of LGT proteome, a potential biomarker of severe TBI, in serum of severe TBI patients. The LGT proteome presents in the serum of severe TBI patients. The abundance diversity of LGT proteome is closely associated with pathologic condition of TBI patients. Serum LGT proteome may be used as a promising marker for evaluating severity of severe TBI.

## 1. Introduction

Traumatic brain injury (craniocerebral injury, TBI) is the most common disease with high morbidity and mortality in neurosurgery, especially to the youngsters, and a lot of researchers have focused on this fact [1, 2]. Severe TBI represents a major cause of neurological morbidity and mortality throughout the world. In the past decades, several challenges have been faced in the conduct of clinical research in TBI, including inclusion of a broad heterogeneity of injuries, difficulties with standardization and consistency of complex medical management, and lack of sophisticated measures to sufficiently detect the differences in outcomes. Recent developments in definition of potential genetic and biological markers in TBI are also aiding in the subcategorization of patients into finer diagnostic and prognostic groups [3].

Serum protein biomarkers have long held promise in the study of TBI. They are considered to facilitate the diagnosis, monitoring progress, prognosis, and providing pertinent molecular information about ongoing pathological changes for designing evidence-based therapeutic interventions [4]. Serum protein biomarkers are of special importance in TBI because they are typically associated with military operations with limited access to imaging and other diagnostic tools of hospitals [5, 6]. So there is great significance to look for the related protein markers to the TBI patients' pathogenetic condition and prognosis [2, 7]. Pei [8–10] reported a kind of proteome called lost goodwill target (LGT) proteome in the serum of the tumor patients which can reflect the different phase of tumor development or aggravation, which was also related to the patients' pathogenetic condition and mortality. Ren et al. [11] reported that the LGT proteome was produced under the pathologic condition of TBI patients, and the abundance of LGT proteome is closely associated with pathogenetic condition and prognosis of TBI patients; the LGT proteome may play an important role in predicting pathogenetic condition and prognosis of TBI patients.

Based on the findings above, LGT can also be found in severe TBI patients' serum, and it may be the early warning sign for disease aggravation or even death. In order to investigate the clinical significance of LGT proteome, the study proceeded with surface enhanced laser desorption/ionization time-of-flight mass spectrometry (SELDI-TOF-MS) [12, 13] technology, which can detect the exact LGT concentration in the serum of TBI severe patients.

## 2. Materials and Methods

### 2.1. General Data

96 TBI cases treated in our department between March 2006 and July 2009 were analyzed retrospectively. There were 77 males and 19 females with age range of 16–83 years (mean age: 31.7 ± 26.3); interval time from injury to clinical (h): 0.5~11.5 h, average 7.1 ± 2.2; Glasgow Coma Scale (GCS) score: 44 cases with 3~5 points, 52 cases with 6~8 points, and 32 cases receiving emergency operation; trauma cause: 46 cases by traffic accident, 22 cases by injury due to fall or mine disaster, 19 cases by strike injury, and 6 cases with other causes; major trauma type: 47 cases with contusion and laceration of brain and (or) subdural/intracerebral hematoma, 14 cases with epidural hematoma, and 35 cases with diffuse axonal injury and brain stem injury. All cases were diagnosed as craniocerebral trauma by cranial CT scanning after the admission; 69 survival cases carried on prognostic evaluation with Glasgow outcome score (GOS) 6 months later. The exclusion criteria were as follows: being under 16 years old, death in 24 hours from admission, severe infection or combined injury, malignant tumor or autoimmune disease, with damage of important organs especially liver and kidney (multiple organ failure), and transfusion of blood in operation. Meanwhile, 35 healthy cases were chosen as control group. This project has been reviewed by the Medical Ethics Committee of Shanxi Provincial People's Hospital. At the same time, signed informed consent of research projects of every patient with patients themselves or their guardians was obtained. The factors such as general condition, age, and sex were comparable (in research group and the normal controls) between the two groups (*P* > 0.05).

### 2.2. SELDI-TOF-MS Detection

#### 2.2.1. Main Instruments and Reagents

The main instruments and reagents included protein fingerprint meter, WCX2 chips, PBS-IIc chip reading machine, and Ciphergen ProteinChip 3.2 analysis software, urea, sodium acetate [NaAc (100 mmol/L NaAc pH 4.0)], HPLC water, and SPA.

#### 2.2.2. Serum Sample Processing

5 mL sera were taken in the morning when patients were hollow and were put in VACVTTE vacuum quantitative mining container and placed at room temperature for 60 min (including the time when samples were out of the body), and then the serum was separated from blood at 3000 g × 10 min and preserved at −80°C; serum samples were thawed and put in ice bath (10000 g × 2 min); take 5 *μ*L serum sample and add 10 *μ*L urea (9 mmol/L). The sample underwent oscillation for 30 min, followed by the addition of 180 *μ*L buffer (100 mmol/L NaAc, pH = 4.0), at 4°C, and was centrifuged at 10000 g × 2 min; then the supernatant fluid was taken for use.

#### 2.2.3. Chip Processing

Chip was put into the container with hydrochloric acid (100 mmol/L), oscillated for 4~5 min, and then washed with HPLC water; put the chip into the bioprocessor: start with each hole plus 200 *μ*L combined cushioning liquid and carry out oscillation for 5 min at room temperature, discard liquid, and then repeat the procedure; then continue with each hole plus 100 *μ*L serum sample at room temperature and carry out oscillation for 1 h. Discard the liquid and wash the hole with 200 *μ*L combining buffer 2 times (5 min each time) and then with HPLC water. Dismantle the chip, perform natural drying, and add 0.5 *μ*L SPA at each point, twice. Read the meter reading chip with WCX2 protein microarray after drying.

#### 2.2.4. Data Collection

The same parameters were used for all the samples; data were collected using Ciphergen ProteinChip 3.2 analysis software. Set inspection sample laser intensity at 195, detection sensitivity at 8, collected data quality load ratios (*M*/*Z*) in the range 1.0 × 10^3^~2.0 × 10^3^, and collected position 20~80, with each point collected 20 times; the total points were 140 times. Before each collection, all-in-one protein microarray calibration instrument was used to reduce the error to less than 0.1%. Using the quality control protein array to do the repetitive detection, the peak and strength variation coefficient (SVC) are controlled below 0.05% and 15%, respectively.

#### 2.2.5. Observing Indexes

In the fingerprint, qualitative load ratios (*M*/*Z*) between 11100 + HDa and 11900 + HDa exhibit cluster sample of fingerprint which is LGT existing performance. Observe LGT proteome abundance values and the LGT dynamically in trauma group and control group.

### 2.3. Statistical Analysis

All measurement data was represented with mean ± standard deviation (*x* ± SD), and multiple comparisons of the average number of diversity samples were examined through SNK-*q* test; the count data was examined through *χ* ^2^ test. SPSS11.0 was used for statistical analysis. *P* < 0.05 was considered statistically significant.

## 3. Results

### 3.1. LGT Proteome Expression in TBI Patients

**Figure 1:**
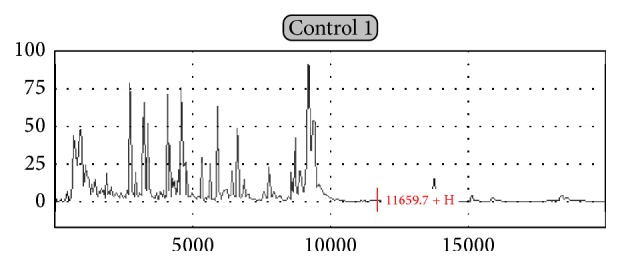
LGT proteome does not present in the protein fingerprint spectra of normal controls.

**Figure 2:**
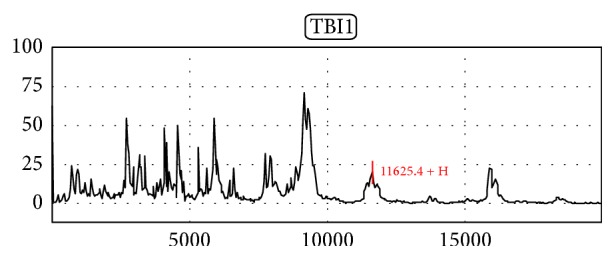
LGT proteome presents in protein fingerprint spectra of TBI group.

The expression of LGT proteome was *M*/*Z* between 11100 + HDa and 11900 + HDa, showing a group of three peaks or three peaks above cluster of fingerprint. The comparison between the upstream and downstream peak cluster indicated the *M*/*Z* difference of above 1000 quality units, eye-catching background, and outstanding fingerprint characteristics. Take the peak more than 5% for LGT proteome positive diagnosis standard [8–10]. In this study, TBI patients showed significant LGT proteome expression patterns (Figure 2), while the control group did not present LGT protein expression spectrum (Figure 1).

### 3.2. The Comparison of Early Results after Admission among the GCS 6–8 Severe TBI Group, GCS 3–5 Severe TBI Group, and the Control Group (Table 1)

On the first day within 24 hours of admission, we collected the first serum specimen to detect the differences of TBI patients' LGT proteome abundance values. The result shows that the LGT proteome abundance of the GCS 6–8 severe TBI group and GCS 3–5 severe TBI group was obviously higher than of the control group; the LGT proteome abundance of the GCS 3–5 severe TBI group was significantly higher than of the GCS 6–8 severe TBI group (Table 1).

### 3.3. LGT Proteome Results in Different Types of Craniocerebral Trauma

According to the different types of TBI, all the cases were assigned into ① brain stem injury and diffuse axonal injury, ② contusion and laceration of brain and (or) subdural/intracerebral hematoma, and ③ epidural hematoma and/or skull fractures, analyzing three groups of LGT proteome abundance values. Results showed that the LGT proteome abundance values of groups ① and ② were significantly higher than of group ③, and the difference was statistically significant (*P* < 0.01), but there were no significant differences between groups ① and ② (Table 2).

### 3.4. LGT Proteome Dynamic Change (Comparison with Death Group and Live Group)

LGT proteome expression was detected in two groups of patients in the whole procedure; death group was higher than the survival group in the course of different stages; then, the abundance values of two groups showed a gradual decline rather than a progressive manner. Besides, a moderate elevation was shown in both groups on the seventh day. During the early phase (days 1 and 4), LGT proteome abundance value of death group was significantly higher than of the survival group (*P* < 0.05). With the progression of the course (days 7 and 14), there was no significant difference between the two groups (*P* > 0.05) (Table 3).

## 4. Discussion

Traumatic brain injury (TBI) is the leading cause of death and disability among young adults [2]. Severe TBI is associated with a 30%–70% mortality rate [14]. The low sensitivity and specificity of current indexes used to assess the injury degree and prognosis (GCS score, pupil reflection) and imaging (CT) contribute to the high morbidity and mortality of TBI [2, 15, 16]. Finding specific biomarker of proteome will provide a new method to explore the nosogenesis and prognosis of TBI [7]. Proteome is the biomarker of TBI [17, 18]. The research on the relationship between the dynamic change of serum and the injury degree/prognosis of TBI was common; it often contained neuron-specific enolase (NSE) [2, 15, 19–22], S100B (S-100*β*) [2, 15, 22–25], BB isozyme of creatine kinase (CK-BB) [2, 15, 26], glial fibrillary acidic protein (GFAP) [2, 15, 22, 27], myelin basic protein (MBP) [2, 15], nerve growth factor (NGF) [20], doublecortin (DCX) [20], high-mobility group box 1 (HMGB1) [28], and so forth. After severe TBI, authors of several studies have studied biomarkers in the acute phase; biomarkers often contained S100B [14, 29, 30], ubiquitin carboxy-terminal hydrolase L1 (UCH-L1) [31, 32], glial fibrillary acidic protein (GFAP) [32], Interleukin-8 (IL-8) [33], Interleukin-10 (IL-10) [34], tumor necrosis factor *α* (TNF-*α*) [33], cell-free DNA (cf-DNA) [35], and so forth. However, the correlation between structural damage and these biomarkers has not been elucidated; it was difficult to find a serum biomarker with high specificity to assess the injury degree and prognosis of severe TBI.

Recently, Pei et al. [8–10] found that the serum concentration of LGT proteome was related to the mortality of tumor patients through 146 cases; the mortality was 100% in the group which has persistent positive LGT proteome concentration and 0% in the group which was negative. If LGT proteome is continuously negative, it means that the disease lies stable. On the contrary, if LGT proteome is continuously positive, it implies disease progression and indicates poor prognosis. Taken as a diagnosis platform to predict tumor progressing and by conducting corresponding treatment, it helps to extend the survival period of patients, so LGT proteome was a promising alert proteome which could forecast the prognosis of tumor patients. Negative LGT proteome fingerprint in the serum of patients with malignant tumors could be viewed as the stationary phase, while the positive LGT proteome could be viewed as the exacerbation stage. When LGT proteome turns from negative to positive, it could be viewed as a sign of progression, while transition from positive to negative indicates remission. The LGT proteome closely correlated with the severity of disease condition and prognosis in patients with critical illness [36]. The LGT proteome presents in the serum of TBI patients, and it is a proteome that appeared under pathologic state [37]. We presume that LGT proteome may also exist in the serum of severe TBI patients and could be prewarning for the aggravation and the mortality of severe TBI.

Identification and purification of candidate biomarkers is a critical step in the biomarker development process, since it provides insight into the disease biology and facilitates the development of analyte-specific assays [38]. Technical problems include selecting the best proteomics method for serum biomarker discovery [5]. Potential proteomic biomarkers can be notably determined through SELDI-TOF-MS [39, 40]. SELDI-TOF-MS is a widely used technique for diagnostic biomarker discovery in plasma, serum, and tissue and has the ability to carry out screening and identification. During the last years, this technique has allowed high throughput and simplicity of experimental procedures to become an important research tool for biomarker discovery [12, 13]. The characteristic of LGT proteome is the appearance of a group of three peaks or three peaks above cluster of fingerprint at *M*/*Z* between 11100 + HDa and 11900 + HDa. One of the spectrum abundance values was ≧20%, but all abundance values were ≧5%, compared with the upstream and downstream peak cluster; the *M*/*Z* values were more than 1000 quality units, with striking background as well as outstanding fingerprint characteristics. So take the peak more than 5% for LGT proteome positive diagnosis standard [8–10].

The experiment proceeded through SELDI-TOF-MS technology; the LGT proteome was detected in the proteome fingerprint with 100% relative abundance. A fingerprint marker just like a cluster appeared between 11100 Da and 11900 Da *M*/*Z*; the upstream cluster peak got more than 1000 mass units (*M*/*Z*) than downstream cluster peak; it showed the same result as the finding of Pei [8–10], so it proved that the LGT proteome could be detected in the serum of TBI patients. The LGT proteome was not detected in the serum of the controls. The abundance of LGT proteome of GCS 6–8 severe TBI group and GCS 3–5 severe TBI group was significantly higher than of the controls (*P* < 0.01). The abundance and positive rate of LGT proteome of GCS 3–5 severe TBI group were significantly higher than of GCS 6–8 severe TBI group (*P* < 0.01). Decreased serum LGT level is associated with GCS scores. The results showed that the LGT proteome could be detected in serum of TBI patients; the abundance of LGT proteome is closely associated with pathogenesis of TBI patients. TBI often involves a combination of mechanical trauma and local hypoxemia, on which serum biomarker concentrations may provide critical therapeutic data to evaluate the brain injury. Serum biomarker after TBI could be used as a source for the identification of the brain injury, the extent of the injury, and the time of its occurrence and even for identifying its most likely outcome [41].

Our present study divided the TBI patients into three groups: ① brain stem injury and diffuse axonal injury, ② contusion and laceration of brain and (or) subdural/intracerebral hematoma, and ③ epidural hematoma and (or) skull fracture. The result showed that the LGT proteome abundance in groups ① and  ② was significantly higher than in group ③ (*P* < 0.01), but it did not reveal a significant difference between group ① and group ②. The result showed that the LGT proteome had close relationship with the severity of craniocerebral trauma. Neuronal and glial cell loss results from necrotic and apoptotic cell death during the primary and secondary injury and may lead to an increase in various neuron- and glia-specific proteins in serum [42]. Elevated serum levels of neuron- or glia-specific proteins indicate increased permeability of the blood-brain barrier (BBB, in addition to neuronal and glial cell damage or loss) [33]. Severe TBI not only causes substantial direct tissue damage, but also instantaneously breaks down existing biological barriers, generating massive pathological responses (e.g., metabolic changes) to toxic molecules and cellular debris. Metabolic changes include hypoxia, altered cell metabolism (e.g., glucose utilization), disrupted energy levels and utilization (leading to ionic imbalance, excitotoxicity, etc.), enhanced inflammatory activity, and increased systemic hormonal secretion [43, 44]. To our best knowledge, the various forms of severe TBI can share common pathological “components” during both the primary and the secondary injury processes. There was no significant difference in the degree of injury between group ① and group ②, which was consistent with their clinical symptoms due to the insignificant difference of the degree of injury between severe contusion and laceration of brain and brain stem injury or diffuse axonal injury.

The experiment also analyzed the LGT proteome abundance during the dynamic change of severe TBI patients. The LGT proteome abundance was higher in death group than in survival group in the initial pathologic stage and then decreased in the two groups as the course was prolonged. At the early pathologic stage (days 1 and 4), the LGT proteome abundance was significantly higher in death group than in survival group (*P* < 0.05); there was no significant difference between the two groups in days 7 and 14 (*P* > 0.05). So the LGT proteome abundance at the early pathogenetic stage could be used to predict the different prognosis of TBI patients; the higher the LGT proteome abundance was, the worse the prognosis was. The experiment also showed that the LGT proteome abundance of death and survival groups increased in day 7. It happened during the secondary craniocerebral injury period, so the increase of the LGT proteome abundance may be caused by the secondary craniocerebral injury. There is a second wave of long-lasting pathological changes called the secondary injury mechanism. These pathologic features include metabolic changes, neuroinflammation, vascular abnormalities, and neuronal and glial cell death [45, 46]. The temporal aspects of injury, like the onset of the various secondary pathologic features (cyto- and vasogenic factors leading to edema, vasospasm, and altered rates of perfusion), are especially important in dynamically changing diseases like severe TBI. This study illustrates how to monitor the temporal pattern of changes (e.g., “time to peak”) of serum LGT levels, which can be useful for identifying injury severity and outcome. We can state that the determination of LGT levels in serum could act as a biomarker for the early prediction of mortality after severe TBI.

## 5. Conclusion

This is the first study to investigate the biokinetics of LGT proteome, a potential biomarker of severe TBI, in serum of severe TBI patients. The LGT proteome presents in the serum of severe TBI patients. The abundance diversity of LGT proteome is closely associated with pathologic condition of TBI patients. Serum LGT proteome may be used as a promising marker for evaluating severity of severe TBI. Further studies of LGT proteome are needed to validate these findings in a larger scale of samples and should be conducted to support these associations with outcomes.

## The Innovation

Serum LGT proteome was detected by surface enhanced laser desorption/ionisation time-of-flight mass spectrometer (SELDI-TOF-MS). The LGT proteome presents in the serum of severe TBI patients. The abundance diversity of LGT proteome is intimately associated with pathogenetic condition of TBI patients. Serum LGT proteome may be used as a promising marker for evaluating severity of severe TBI.

## Figures and Tables

**Table 1:** The LGT proteome abundance comparison in the GCS 6–8 severe TBI group, GCS 3–5 severe TBI group, and normal controls.

Group	Cases (*n*)	Positive proportion	Positive rate (%)	LGT proteome abundance (%)
GCS 3–5 severe TBI group	44	37/44	84.09^▲^	17.37 ± 6.87^△▲^
GCS 6–8 severe TBI group	52	21/52	38.46	8.39 ± 5.38^△^
Normal controls	35	0/35	0	1.79 ± 0.81

^△^Significant difference to normal controls (*P* < 0.01). ^▲^Significant difference to severe brain injury group (*P* < 0.01).

**Table 2:** The analysis of the LGT proteome abundance in different TBI groups.

Group	Cases (*n*)	LGT proteome abundance
①: brain stem injury and diffuse axonal injury	35	15.68 ± 10.73^▲^

②: contusion and laceration of brain and (or) subdural/intracerebral hematoma	47	14.16 ± 9.89^▲△^

③: epidural hematoma and (or) skull fracture	14	5.97 ± 5.13

^▲^Compared with group ③ (*P* < 0.01). ^△^No significant difference with group ② (*P* > 0.05).

**Table 3:** The Comparison of LGT proteome abundance in different pathogenetic stage.

Prognosis		Day 1	Day 4	Day 7	Day 14
Death group	Cases LGT abundance	29 26.09 ± 10.92	23 15.13 ± 5.79	16 17.01 ± 13.94	9 11.92 ± 4.99

Survival group	Cases LGT abundance	67 8.98 ± 6.73	67 6.07 ± 4.89	67 10.53 ± 13.68	67 5.62 ± 7.07

*P* value		<0.01	<0.05	>0.05	>0.05
