# Corrigendum to “Determination of Serum Lost Goodwill Target Proteome in Patients with Severe Traumatic Brain Injury”

**DOI:** 10.1155/2018/5234910

**Published:** 2018-08-23

**Authors:** Hongming Ji, Changchen Hu, Gangli Zhang, Jinrui Ren, Yihu Tan, Wenxiao Sun, Junwen Wang, Jun Li, Hongchao Liu, Ruifan Xie, Zhipeng Hao, Dongsheng Guo

**Affiliations:** ^1^Department of Neurosurgery, Shanxi Provincial People's Hospital, Taiyuan 030001, China; ^2^Department of Neurosurgery, Tongji Medical College, Huazhong University of Science and Technology, Wuhan 430030, China; ^3^Department of Neurosurgery, Liyuan Hospital, Tongji Medical College, Huazhong University of Science and Technology, Wuhan 430077, China; ^4^Department of Neurosurgery, Wuhan Central Hospital, Tongji Medical College, Huazhong University of Science and Technology, Wuhan 430014, China

In the article titled “Determination of Serum Lost Goodwill Target Proteome in Patients with Severe Traumatic Brain Injury” [1], there was an incomplete description of how the results related to our earlier publications. The predictive value of LGT in TBI was also overestimated. We apologise for this and explain below the relationship between our articles, why we classified the patients as we did, and the use of the grants for this research.

## 1. Previous Articles

Our earlier work was discussed as follows, which was not sufficient: “Ren et al. [11] reported that the LGT proteome was produced under the pathologic condition of TBI patients, and the abundance of LGT proteome is closely associated with pathogenetic condition and prognosis of TBI patients; the LGT proteome may play an important role in predicting pathogenetic condition and prognosis of TBI patients.”

Our three earlier articles also studied serum LGT expression as a potential biomarker in traumatic brain injury. A summary of our four articles is listed in Table 1.

Comparing all types of patients, we believed that the LGT proteome is more important in severe traumatic brain injury (TBI). Thus, in later studies and case collection we focused on severe TBI. Some patients/data were shared between the articles, though there was a different emphasis for each study. The present article [1] contains the final summary of severe TBI.

In the section “The Innovation”, we stated “Serum LGT proteome was detected by surface enhanced laser desorption/ionisation time-of-flight mass spectrometer (SELDI-TOF-MS). The LGT proteome presents in the serum of severe TBI patients. The abundance diversity of LGT proteome is intimately associated with pathogenetic condition of TBI patients. Serum LGT proteome may be used as a promising marker for evaluating severity of severe TBI.” We did not intend to exaggerate the research results, but this overstated the originality of the analysis, as Professor Yi Pei had already used SELDI-TOF-MS technology to detect the LGT proteome [5] and our earlier work using Proteome Dynamic Change found that LGT expression was present in the serum of severe TBI patients. The LGT proteome was found in patients with cancer in Professor Pei's study, but this work studied the abundance of the LGT proteome in critical patients without neoplasms and extended our earlier work on severe TBI.

## 2. Patient Grouping

 The standard classification of TBI on the GCS is severe with 3–8, moderate with 9–12, and mild with 12–14. In this study, the severe TBI group was divided into severe and extraordinarily severe, based on [6, 7] in which severe TBI was divided into two groups, as described in [8]: “The ability of the Glasgow Coma Scale (GCS) to define the severity of traumatic brain injury (TBI), as well as its widespread acceptance and application in neurological assessment of both preclinical and clinical phases, allows for comparison between different studies and provides a sound basis for quality-patient-management in such cases.”

## 3. Survival Analysis

In this study, we investigated the expression of LGT proteome at two weeks and we found that the proteome of patients who later died was increased. Death directly due to brain injury usually occurs within two weeks, and after two weeks it may be attributed to a variety of serious complications and might be unrelated to the primary intracranial lesions. The specific reasons need to be further studied, as the patients' complications and long-term cause of death were not recorded.

## Figures and Tables

**Table 1:** A comparison of the articles by Zhang and colleagues on serum LGT proteome in TBI.

Title	The expression and prognostic of serum LGT proteome in patients with traumatic brain injury [in Chinese] [2]	The Clinical Significance of Changes of the Serum LGT Proteome in Patients with traumatic brain injury [in Chinese] [3]	The alteration of serum LGT proteome in patients with severe traumatic brain injury and its prognostic value [in Chinese] [4]	Determination of Serum Lost Goodwill Target Proteome in Patients with Severe Traumatic Brain Injury [1]
Journal	Chinese Remedies *＆* Clinics	Chinese Remedies *＆* Clinics	Chin J Exp Surg	BioMed Research International

Publication date	February 2011	October 2010	June 2011	July 2015

Enrolled cases	105 cases with TBI	69 cases with TBI	84 cases with severe TBI	96 cases with severe TBI

Period of case collection	March 2006 to July 2008	April 2007 to February 2008	March 2006 to July 2008	March 2006 to July 2009

Patient groups	TBI: GCS 3–8 GCS 8–12 GCS 12–15	TBI: GCS 3–8 GCS 8–12 GCS 12–15	Severe TBI: GCS 3-5 GCS 6-8	Severe TBI: GCS 3-5 GCS 6-8

Research highlights	The factors affecting the surgery, patients in articulo mortis	The relevance of abundance of LGT to prognosis	Abundance of LGT, APACHE II scores and prognosis	The types and locations of TBI, and the survival / outcome of TBI patients

GCS = Glasgow Coma Score.

## Supplementary Materials

Supplementary Materials The original data is included as Supplementary Materials.
