# Supplementary material for: Corrigendum to “Determination of Serum Lost Goodwill Target Proteome in Patients with Severe Traumatic Brain Injury”
Source: Biomed Res Int. 2018 Aug 23;2018:5234910. doi: 10.1155/2018/5234910 (PMC6126061; doi:10.1155/2018/5234910)
Supplement: Supplementary Materials — The original data is included as Supplementary Materials. [file 5234910.f1.zip › Original Data/Data 1/╒┼╕╒└√╩2╛▌/╒┼╕╒└√╩2╛▌/病例血清标本收集表.doc]

**病例血清标本收集表**

| 性别 | 年龄 | 诊断 | 入院GCS评分 | 入院APACHE评分 | 受伤  第1天 | 受伤  第4天 | 受伤第7天 | 受伤  第14天 |
| --- | --- | --- | --- | --- | --- | --- | --- | --- |
| 男 | 18 | 脑干损伤 | 3 | 22 | B004 | B005 | B008 | B009 |
| 男 | 16 | 脑挫裂伤 | 8 | 15 | B006 | B007 | B010 | B012 |
| 男 | 35 | 脑挫裂伤 | 8 | 17 | B013 | B015 | B019 | B021 |
| 男 | 35 | 脑挫裂伤、骨折 | 5 | 19 | B014 | B016 | B020 | * |
| 男 | 50 | 脑挫裂伤、肋骨骨折 | 6 | 21 | B039 | B043 | B046 | B052 |
| 男 | 36 | 弥漫性轴索损伤、SAH | 8 | 16 | B045 | B049 | B051 | B054 |
| 男 | 50 | 脑干损伤、脑挫裂伤 | 5 | 26 | B055 | B058 | B060 | * |
| 男 | 34 | 脑挫裂伤 | 8 | 14 | B056 | B059 | B062 | B065 |
| 男 | 23 | 脑干损伤、SAH | 6 | 19 | B057 | B061 | * | * |
| 男 | 45 | 颅底骨折、前额脑挫裂伤 | 8 | 14 | B072 | B075 | B079 | B083 |
| 男 | 36 | 弥漫性轴索损伤、SAH | 5 | 16 | B074 | B078 | * | * |
| 男 | 62 | 脑挫裂伤、硬膜下血肿 | 8 | 12 | B084 | B089 | B094 | B099 |
| 女 | 56 | 颅底骨折、脑挫裂伤 | 8 | 11 | B087 | B091 | B096 | B100 |
| 男 | 56 | 脑挫裂伤、脑疝、骨折 | 5 | 21 | B088 | B092 | * | * |
| 男 | 43 | 弥漫性轴索损伤 | 6 | 14 | B126 | B129 | B131 | B138 |
|  |  |  |  |  |  |  |  |  |
|  |  |  |  |  |  |  |  |  |

*** 死亡**
