# Supplementary material for: Corrigendum to “Determination of Serum Lost Goodwill Target Proteome in Patients with Severe Traumatic Brain Injury”
Source: Biomed Res Int. 2018 Aug 23;2018:5234910. doi: 10.1155/2018/5234910 (PMC6126061; doi:10.1155/2018/5234910)
Supplement: Supplementary Materials — The original data is included as Supplementary Materials. [file 5234910.f1.zip › Original Data/Data 2/▓╣│Σ╡─╩2╛▌/▓╣│Σ╡─╩2╛▌/病例血清标本收集表(补充).doc]

**病例血清标本收集表(补充)**

| 性别 | 年龄 | 诊断 | 入院GCS评分 | 入院APACHE评分 | 受伤  第1天 | 受伤  第4天 | 受伤第7天 | 受伤  第14天 |
| --- | --- | --- | --- | --- | --- | --- | --- | --- |
| 女 | 76 | 脑挫裂伤 | 14 | 16 | B011 | - | - | - |
| 男 | 31 | 脑干损伤 | 5 | 22 | B041 | B042 | B050 | * |
| 男 | 40 | 脑挫裂伤 | 6 | 23 | B044 | - | - | - |
| 男 | 45 | 脑干损伤 | 6 | 16 | - | B053 | - | - |
| 男 | 35 | 脑挫裂伤 | 7 | 18 | B047 | - | - | - |
| 男 | 34 | 脑挫裂伤 | 7 | 14 | B048 | - | - | - |
| 男 | 29 | 脑挫裂伤 | 5 | 21 | B001 |  |  |  |
| 男 | 25 | 脑干损伤 | 4 | 25 | B002 | B003 |  |  |
| 男 | 60 | 硬膜下血肿 | 4 | 23 | B063 | * | * | * |
| 男 | 66 | 弥漫性轴索损伤 | 3 | 27 | B064 | B066 | - | B068 |
| 男 | 31 | 脑干损伤 | 3 | 28 | B069 | B070 | * | * |
| 男 | 21 | 脑挫裂伤 | 4 | 18 | B076 | - | - | - |
|  |  |  |  |  |  |  |  |  |

*** 死亡**
